# Efficacy and safety of hypofractionated versus conventional radiotherapy in breast cancer: a systematic review and meta-analysis

**DOI:** 10.3389/fonc.2026.1779983

**Published:** 2026-05-13

**Authors:** Malek Talal, Marafi Jammaa Ahmed, Muhammad M. Elsharkawy, Alaa R. AL-Ihribat, Mohamed F. Srour, Youssef Z. Farhat, Muhammad Youssef, Rabeia Babiker Mustafa, Mohamed A. Wafa, Ahmed Werdany Hassan, Aya Ahmed Shimal, Omran Shrebaty, Ibrahim Moqbel

**Affiliations:** 1 Faculty of Medicine, Minia University, Minia, Egypt; 2 Faculty of Medicine, University of Bahri, Khartoum, Sudan; 3 Alexandria Faculty of Medicine, Alexandria, Egypt; 4 College of Medicine and Health Sciences, Palestine Polytechnic University, Hebron, Palestine; 5 Faculty of Medicine, Menoufia University, Shebin El Kom, Egypt; 6 Misr University for Science and Technology, Cairo, Egypt; 7 Faculty of Medicine, Tanta University, Tanta, Egypt; 8 Faculty of Medicine, Nile Valley University, Atbara, Sudan; 9 Faculty of Medicine, Mansoura University, Mansoura, Egypt; 10 College of Medicine, University of Baghdad, Baghdad, Iraq; 11 College of Medicine, Sulaiman Al Rajhi University, Al Bukayriyah, Saudi Arabia; 12 Faculty of Medicine, Cairo University, Cairo, Egypt

**Keywords:** breast cancer, disease-free survival, hypofractionated radiotherapy, conventional fractionation, whole-breast irradiation (WBI), acute radiation dermatitis, lymphedema, meta-analysis

## Abstract

**Background:**

Breast cancer is one of the most frequent cancers globally, and radiotherapy plays an important key role in reducing the chance of recurrence following breast-conserving surgery. As an alternative to traditional fractionated radiotherapy (CFRT), hypofractionated whole-breast irradiation (HFRT) has gained popularity since it provides similar oncological results over shorter treatment periods. Nevertheless, the comparative effectiveness and safety profile of hypofractionated versus conventional fractionation schedules remains an area of ongoing evaluation.

**Aim:**

To compare the efficacy and safety of HFRT versus CFRT in patients with breast cancer, with particular emphasis on disease-free survival and treatment-related toxicity.

**Methods:**

A comprehensive search of PubMed, Cochrane Library, Scopus, Web of Science, and Embase was conducted through December 2024. Randomized controlled trials (RCTs) and comparative studies evaluating HFRT versus CFRT in early-stage breast cancer. Key outcomes assessed included disease-free survival (DFS) and treatment-related toxicities, particularly lymphedema and acute radiation dermatitis. The Mantel–Haenszel method to calculate risk ratios (RRs) with 95% confidence intervals (CI) was used.

**Results:**

Eight studies comprising 5,495 breast cancer patients were identified. The meta-analysis demonstrated no significant differences in disease-free survival (DFS) in 5 years (*RR = 1.01, 95% CI 0.96–1.06; P = 0.83*). Similarly, no significant difference was observed in the incidence of lymphedema between HFRT and CFRT (RR = 1.19, 95% CI 0.94–1.51; P = 0.15). Regarding acute radiation dermatitis, a statistically significant difference favoring HFRT was observed under the fixed-effects model (RR = 1.62, 95% CI 1.20–2.18; P = 0.002). However, due to substantial heterogeneity across studies (I² = 90%), a random-effects model was applied, which demonstrated no statistically significant difference between treatment groups (RR = 1.43, 95% CI 0.51–4.01; P = 0.50). These results should be interpreted with caution given the high between-study variability.

**Conclusion:**

HFRT demonstrated comparable oncological outcomes to CFRT, with a trend toward reduced acute skin toxicity, indicating that it could be a promising therapy option for early-stage breast cancer. Standardizing radiation treatments and evaluating long-term toxicity and patient-reported results should be the goals of future research. Also, the observed variability across studies warrants cautious interpretation of the pooled estimates, particularly for outcomes demonstrating substantial heterogeneity.

**Systematic Review Registration:**

https://www.crd.york.ac.uk/PROSPERO/, identifier CRD42025631012.

## 1 Introduction

Breast cancer is one of the most common malignancies worldwide and continues to represent a major cause of cancer related morbidity and mortality among women (1, 2). Over the last ten years, survival rates have grown dramatically due to advancements in multidisciplinary treatments, including surgery, hormonal therapy, endocrine therapy, and radiotherapy, as well as increases in early detection through screening (2). Whole-breast irradiation (WBI) following breast-conserving surgery (BCS) is an important component of treatment for early-stage breast cancer, reducing the risk of local recurrence and breast cancer-related mortality (3).

Currently, hypofractionated radiation is the standard of care for whole-breast irradiation, with shorter regimens demonstrating equivalent local control and toxicity compared with conventional fractionation. Randomized trials have shown that hypofractionated schedules provide outcomes comparable to standard 25-fraction treatment while shortening treatment duration (4–6).

Although hypofractionation has been widely adopted as the standard of care, variation persists in clinical practice regarding fractionation schedules, and the comparative effectiveness and safety profiles across different patient populations remain an area of ongoing evaluation. While previous meta-analyses have compared hypofractionated and conventional fractionation schedules, many differed in clinical focus, scope, or included datasets, and did not comprehensively evaluate contemporary outcomes across the full spectrum of toxicities and survival endpoints. Therefore, an updated and focused synthesis of current evidence comparing HFRT versus CFRT is clinically warranted.

In this study, we conducted a systematic review and meta-analysis to compare hypofractionated and conventional fractionated radiotherapy in patients with early-stage breast cancer, with a focus on disease-free survival and treatment-related toxicities.

## 2 Methods

This study followed a systematic review and meta-analysis design, guided by the Cochrane Handbook for Systematic Reviews of Interventions (7). and the Preferred Reporting Items for Systematic Reviews and Meta-Analyses (PRISMA) 2020 statement (8). The review was registered in PROSPERO (CRD42025631012).

### 2.1 Study selection and eligibility criteria

Studies were considered eligible if they met the following inclusion criteria:

#### 2.1.1 Population

Adult patients with breast cancer receiving radiotherapy following surgery.

#### 2.1.2 Intervention

Hypofractionated radiotherapy.

#### 2.1.3 Comparator

Conventional fractionated radiotherapy.

#### 2.1.4 Outcomes

At least one of the following outcomes was reported: disease free survival or treatment related toxicity, including lymphedema, acute radiation dermatitis, infection, or radiation pneumonitis.

#### 2.1.5 Study design

Randomized controlled trials (RCTs) and comparative cohort studies, including both retrospective and prospective designs.

We excluded non-breast cancer populations, non-surgical cohorts, non-peer-reviewed articles, conference abstracts, and protocols without quantitative outcomes or with incomplete data.

### 2.2 Literature search strategy

A comprehensive search was conducted across the following databases: PubMed, Cochrane Library, Scopus, Web of Science, and Embase through December 19, 2024. The search strategy combined Medical Subject Headings (MeSH) and free-text terms related to breast cancer and radiotherapy fractionation. Key search terms included: ‘breast cancer’, ‘breast neoplasm’, ‘hypofractionated radiotherapy’, ‘hypofractionation’, ‘conventional fractionation’, ‘standard fractionation’, ‘whole-breast irradiation’, and ‘radiotherapy’. These terms were combined using Boolean operators (‘AND’, ‘OR’) to optimize sensitivity and specificity. Reviews, editorials, and case reports were excluded to ensure inclusion of only primary data studies.

### 2.3 Screening and study selection

A two-stage screening process was implemented. In the first stage, two independent reviewers screened titles and abstracts for relevance. In the second stage, the full texts of potentially eligible studies were reviewed independently by the same reviewers. Any disagreements were resolved through discussion or by consulting a third senior reviewer (IM.).

### 2.4 Data extraction

Three reviewers independently extracted data using a standardized electronic form. Extracted variables included:

#### 2.4.1 Study characteristics

Author name, year of publication, country, and study design.

#### 2.4.2 Treatment data

Radiotherapy protocols, fractionation details.

#### 2.4.3 Patient data

Age and baseline demographics.

#### 2.4.4 Outcomes

Disease free survival and treatment related toxicities, including lymphedema and acute radiation dermatitis.

Disagreements were resolved by re-checking the original sources and through consensus with the senior author (IM). Data were organized into structured tables for consistency and ease of analysis.

### 2.5 Risk of bias assessment

Risk of bias in observational studies was evaluated using the ROBINS-I tool, which assesses seven domains, including confounding, participant selection, and outcome measurement (9).

For randomized trials, the Risk of Bias 2 (RoB 2) tool was used to evaluate domains such as randomization process, deviations from intended interventions, and reporting bias.

Two independent reviewers conducted the assessments (10). Discrepancies were resolved by discussion or arbitration with the senior reviewer.

### 2.6 Statistical analysis

Statistical analyses were conducted using Review Manager (RevMan) software, version 7.2.0. While categorical outcomes were assessed using the Mantel–Haenszel method to calculate risk ratios (RRs) with corresponding 95% confidence intervals (CIs). A random-effects model was applied when significant heterogeneity was detected among studies, whereas a fixed-effects model was used in cases of low heterogeneity. Heterogeneity was evaluated using the Chi-square (χ²) test and the I-squared (I²) statistic. I² values were interpreted as follows: 0–40% indicating low heterogeneity, 30–60% moderate heterogeneity, and 50–90% substantial heterogeneity. A p-value of less than 0.10 for the Chi-square test was considered indicative of statistically significant heterogeneity. The results of the meta-analysis were visually presented using forest plots.

### 2.7 Publication bias

Due to the inclusion of fewer than 10 studies in the meta-analysis, formal statistical tests such as Egger’s regression were not performed. However, the potential for publication bias cannot be excluded and may influence the results.

## 3 Results

### 3.1 Study selection

**Figure 1:**
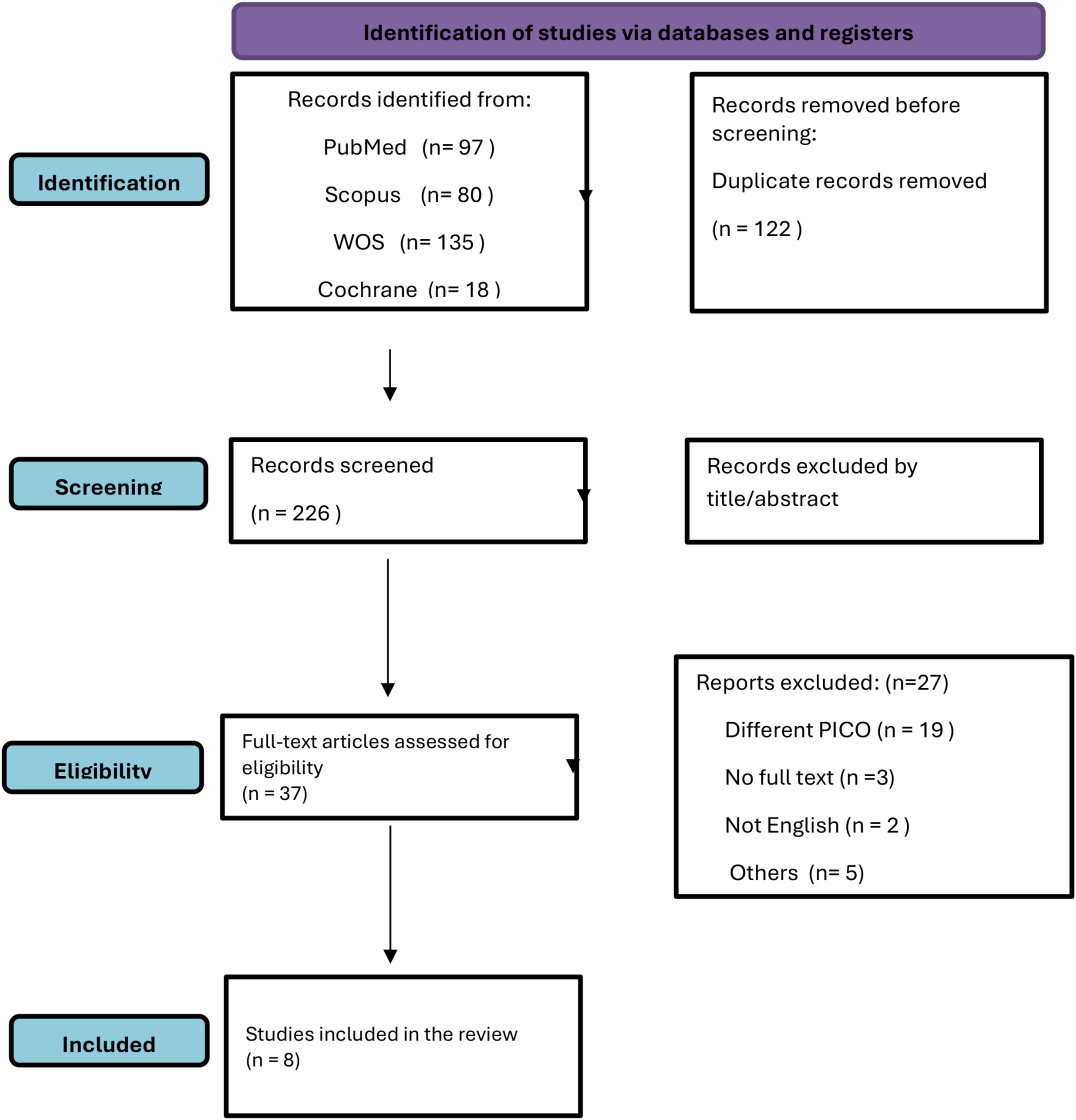
The flow diagram of the study selection process shows the identification, screening, eligibility, and inclusion stages according to PRISMA guidelines.

The flow diagram for the study selection process is shown in **Figure 1**. The search, covering the period from 2012 to 2024, yielded 348 references. After removing duplicates, 226 references were screened based on titles and abstracts. Of these, 37 full-text articles were assessed for eligibility, resulting in 8 studies being included in systematic review and meta-analysis.

### 3.2 Study characteristics

A summary of the included studies is provided in **Table 1**. The systematic review comprised 8 studies comparing conventional fractionated radiotherapy (CFRT) and hypofractionated radiotherapy (HFRT) in breast cancer patients (11–18). These studies collectively included a total of 5,495 patients, with 5 randomized clinical trials, 2 retrospective cohort studies, and 1 prospective cohort study. The studies were conducted in various countries, including Germany, South Korea, China, Belgium, the USA, Denmark, Egypt, India, and Thailand, as outlined in **Table 1**. All studies focused on breast cancer patients receiving or having completed radiotherapy.

**Table 1:** Summary characteristics of included studies.

Study ID	Country	Study design	Sample size	Follow-up duration	Intervention (HFRT)	Comparator (CFRT)
Rades 2023 (11)	Germany	Retrospective cohort	255	48	40 Gy/15 fx (2.67 Gy/fx) + sequential boost 10 Gy/5 fx (2.0 Gy/fx)	50.4 Gy/28 fx (1.8 Gy/fx) + boost 10 Gy/5 fx (2.0 Gy/fx) or SIB 0.3 Gy/fx
Kim 2021 (12)	South Korea	Retrospective cohort	349	58	44.3 Gy median (range 40.5–48.6 Gy), 2.4–2.7 Gy/fx	50.3 Gy median (range 50–66 Gy), 1.8–2.0 Gy/fx
Wang 2019 (13)	China	Randomized, non-inferiority, open-label, phase 3 trial	810	59.83	43.5 Gy/15 fx (2.9 Gy/fx)	50 Gy/25 fx (2.0 Gy/fx)
Versmessen 2012 (14)	Belgium	Randomized controlled trial	121	26.67	42 Gy/15 fx (2.8 Gy/fx) + SIB 9 Gy/15 fx	50 Gy/25 fx (2.0 Gy/fx) + sequential boost 16 Gy/8 fx
Birgitte V. Offersen et,al 2020 (15)	Denmark	Randomized Phase III Trial	1854	87.12	40 Gy/15 fx (2.67 Gy/fx)	50 Gy/25 fx (2.0 Gy/fx)
Julia S. Wong et,al 2024 (16)	United States	Randomized controlled trial	400	40.4	Hypofractionated radiotherapy	Standard fractionated radiotherapy
Mai Atef et,al 2019 (17)	Egypt	Randomized Prospective Study	66	30	Hypofractionated radiotherapy	Standard fractionated radiotherapy
Chitapanarux 2019 (18)	Thailand	Retrospective cohort	1640	60	40 Gy/16 fx or 45–50 Gy/18–20 fx	50 Gy/25 fx or 56 Gy/28–30 fx

The demographic characteristics of the included patients were predominantly reported, with most studies providing a median or mean age. Detailed demographic data, including age ranges and patient distributions, are presented in **Table 2**. Some studies had incomplete data for certain variables, which is indicated in the summary table where applicable.

**Table 2:** Baseline patients’ characteristics.

Study ID	Study group	Sample size	Age	Female sex n (%)	Grade 3	IDC	ER−	PR−	HER2+	LN	>T2
Rades 2023 (11)	Conventional	127	53% ≤60 y; 47% ≥61 y	127 (100)	34	–	20	20	–	127	40
	Hypofractionated	128	53% ≤60 y; 47% ≥61 y	128 (100)	35	–	20	20	–	128	41
Kim 2021 (12)	Conventional	126	54.8% <45 y; 45.2% ≥45 y	126 (100)	–	107	4	4	9	2	–
	Hypofractionated	223	54.7% <45 y; 45.3% ≥45 y	223 (100)	–	195	1	1	28	0	–
Wang 2019 (13)	Conventional	409	Age ≥50: 202 (49%)	409 (100)	111	381	98	105	111	0	409
	Hypofractionated	401	Age ≥50: 194 (48%)	401 (100)	121	379	101	107	135	0	401
Versmessen 2012 (14)	Conventional	62	58 (11)	62 (100)	16	–	8	17	3	46	24
	Hypofractionated	59	Mean (SD)=55 (11)	59 (100)	12	–	11	13	10	38	20
Offersen et al., 2020 (15)	Conventional	937	59	937	139	123	250	–	63	–	157
	Hypofractionated	917	59	917	164	123	250	–	92	–	136
Wong et al., 2024 (16)	Conventional	201	47	201	–	139	–	–	–	–	15
	Hypofractionated	199	47	199	–	147	–	–	–	–	19
Mai Atef et al., 2019 (17)	Conventional	34	49	34	3	–	6	9	11	–	–
	Hypofractionated	32	54	32	5	–	3	6	7	–	–
Chitapanarux 2019 (18)	Conventional	660	57(49–64)	–	–	–	–	–	–	–	–
	Hypofractionated	980	51(45–58)	–	–	–	–	–	–	–	–

### 3.3 Quality assessment

**Figure 2:**
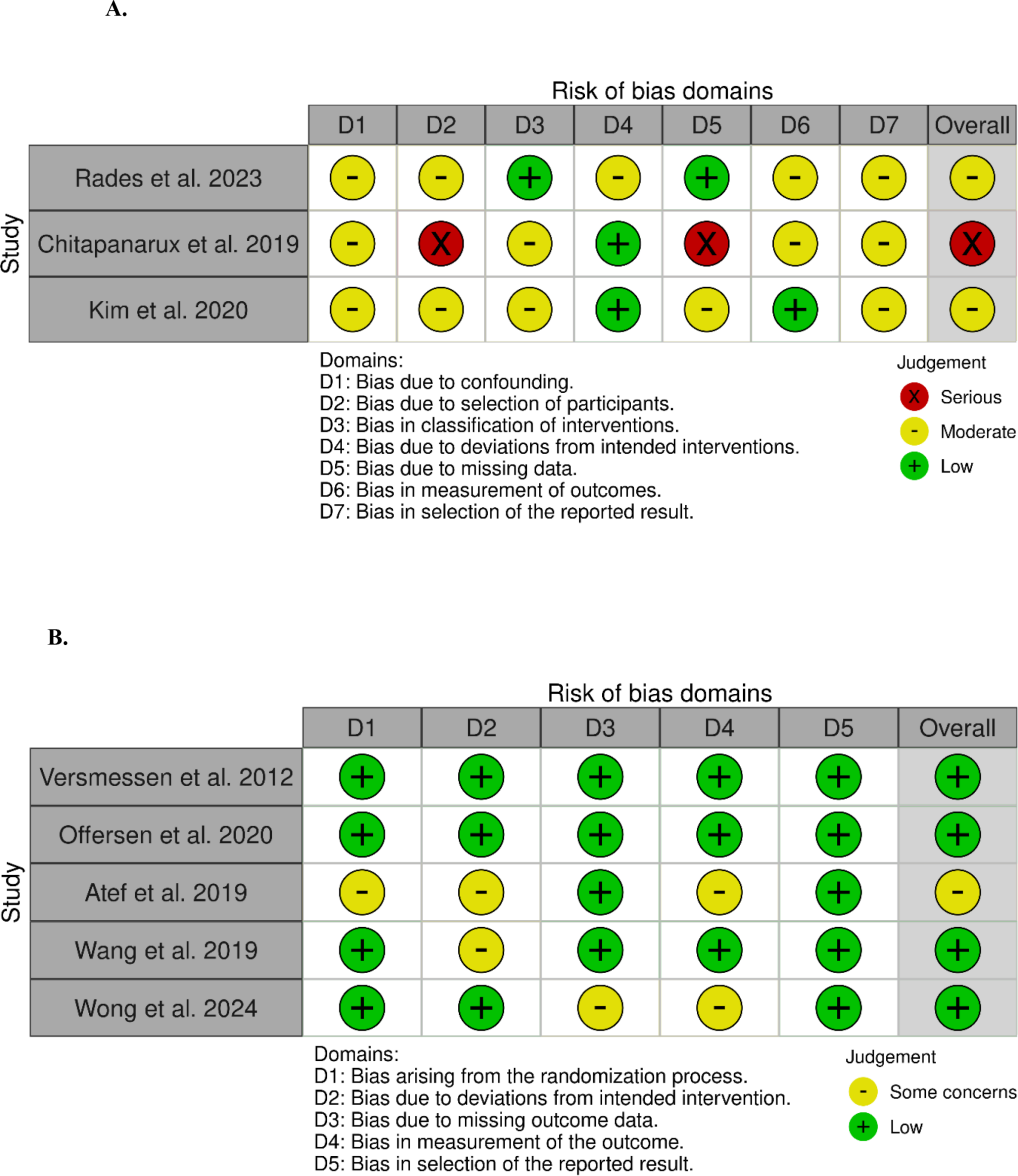
Assessment of risk of bias in the included studies. **(A)** ROBINS-1 evaluation of observational studies. The panel presents a schematic representation of risks (low=green, moderate=yellow) for specific types of biases of each study in the review. **(B)** ROB-2 evaluation of RCTs. The panel presents a schematic representation of risks (low=green, Some concerns=yellow) for specific types of biases of each study in the review.

**Figure 3:**
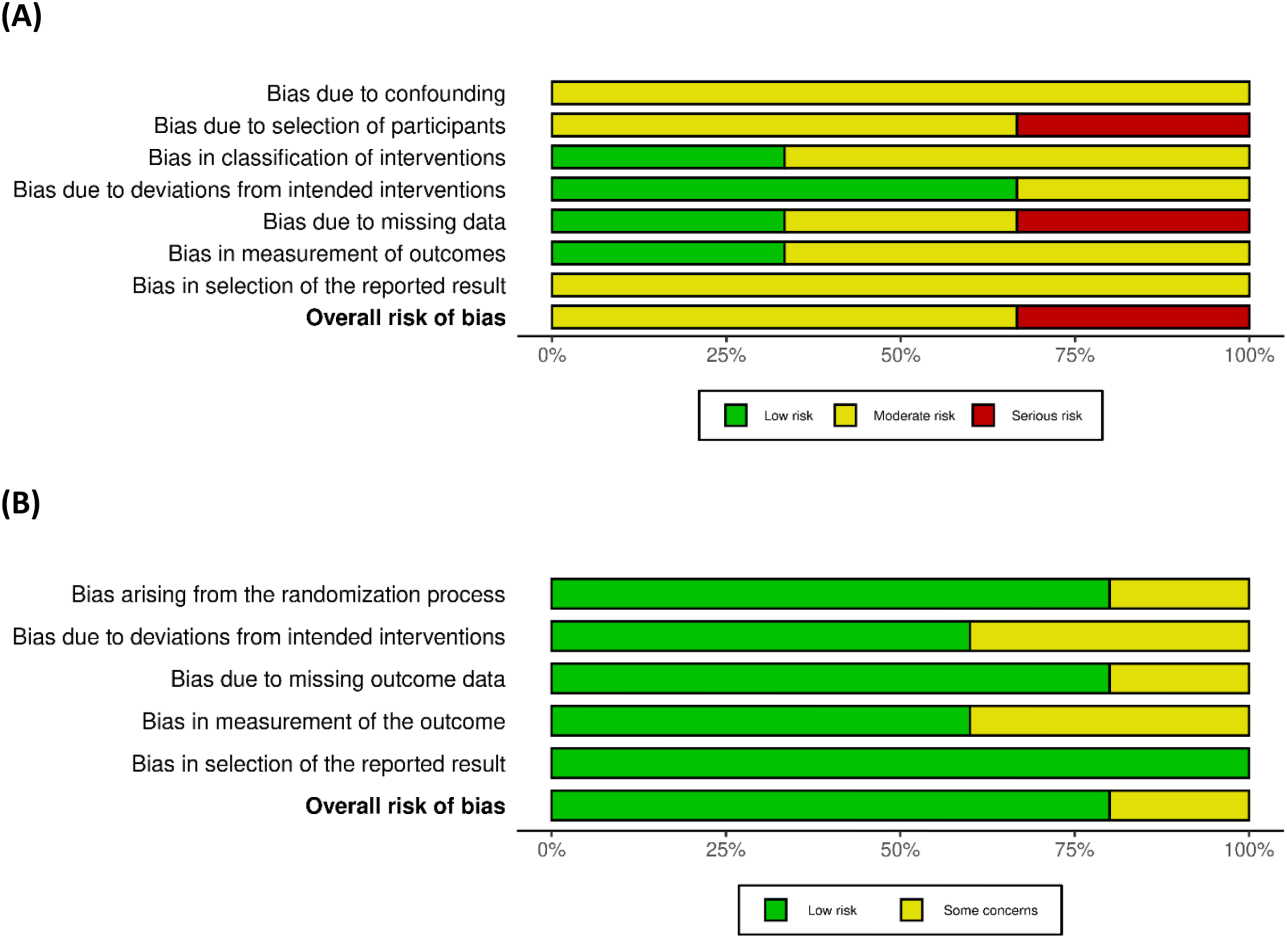
Summary of risk of bias assessment of the included studies. **(A)** ROBINS-1 evaluation of observational studies. **(B)** ROB-2 evaluation of RCTs.

The quality of the included observational studies was assessed using the ROBINS-I tool. Among the two observational studies, both were rated as having moderate risk of bias. The primary sources of bias were related to confounding factors and participant selection. As shown in **Figure 2**, the ROBINS-I domain-level assessment reveals that both observational studies received moderate ratings predominantly in the confounding and participant selection domains, while all other domains were rated as low risk. **Figure 3** provides a summary visualization confirming the predominance of moderate overall risk across these two studies. The five randomized controlled trials (RCTs) were assessed using the ROB-2 tool. Four RCTs were rated as having low risk of bias across all evaluated domains, while one study was classified as having some concerns, with issues concentrated in the domains of deviations from intended interventions and outcome measurement. As illustrated in **Figure 2**, the ROB-2 domain-by-domain assessment confirms that the majority of judgments across all five RCTs were rated as low risk. **Figure 3** presents the overall ROB-2 summary chart, visually confirming the predominance of low risk of bias across the included trials, which supports the reliability of the pooled estimates derived from this meta-analysis.

### 3.4 Outcome measures

Six studies were included in the quantitative analysis. The flow diagram of the study selection process is presented in **Figure 1**, showing the identification, screening, eligibility, and inclusion stages according to PRISMA guidelines.

The outcomes analyzed in the meta-analysis included lymphedema, acute radiation dermatitis, disease-free survival (DFS), comparing conventional fractionation and hypofractionated radiotherapy.

#### 3.4.1 Lymphedema

Four studies comprising 2,798 patients were included in the analysis of lymphedema, with 1,205 patients receiving conventional fractionation and 1,593 receiving hypofractionated radiotherapy. A total of 119 events occurred in the conventional fractionation group and 105 events in the hypofractionated group.

Pooled analysis using the Mantel–Haenszel fixed-effect model demonstrated no statistically significant difference in the risk of lymphedema between conventional and hypofractionated radiotherapy (*RR = 1.19, 95% CI 0.94–1.51*; P = 0.15).

Statistical heterogeneity was low to moderate and not statistically significant (*χ² = 4.81, df = 3, P = 0.19; I² = 38%*).

**Figure 4:**
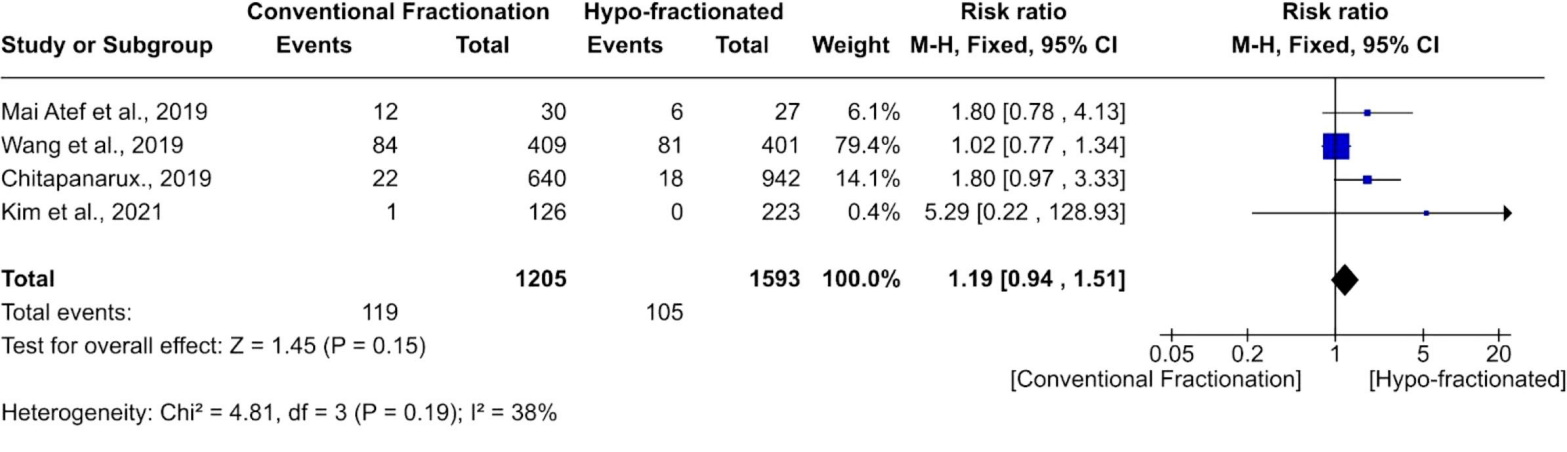
Forest plots illustrate the analysis of lymphedema.

The forest plot (**Figure 4**) shows that the effect estimates from all four included studies are distributed closely around the null value, with overlapping confidence intervals, consistent with the absence of a statistically significant pooled effect and confirming the low-to-moderate heterogeneity (I² = 38%) observed for this outcome.

#### 3.4.2 Acute radiation dermatitis

The pooled analysis using the Mantel–Haenszel fixed-effect model demonstrated a statistically significant difference between treatment groups (*RR = 1.62, 95% CI 1.20–2.18; P = 0.002*). These findings indicate a higher risk of acute radiation dermatitis in the conventional fractionation group compared with the hypofractionated group.

However, substantial statistical heterogeneity was observed across studies *(χ² = 19.30, df = 2, P < 0.0001; I² = 90%*), suggesting considerable variability in effect estimates between trials. Given the presence of substantial heterogeneity across studies, a Mantel–Haenszel random-effects model was applied. The pooled analysis demonstrated no statistically significant difference between treatment groups (*RR = 1.43, 95% CI 0.51–4.01; P = 0.50*).

The wide confidence interval and high I² value indicate considerable between-study variability, and the pooled estimate should therefore be interpreted with caution.

**Figure 5:**
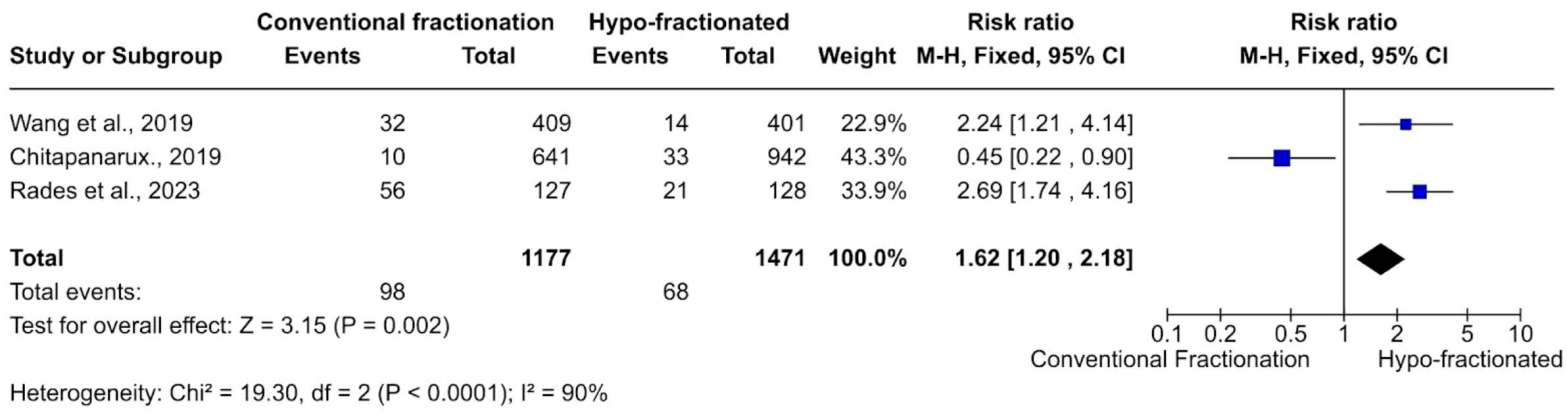
Forest plots illustrate the analysis of acute radiation dermatitis.

**Figure 5** presents the forest plot for acute radiation dermatitis. Under the fixed-effects model, the pooled diamond falls to the right of the null, reflecting the statistically significant result. However, the wide confidence intervals of individual studies and the marked between-study variability visible in the plot visually reinforce the high I² value (90%), supporting the use of a random-effects model. Under the random-effects model, the pooled confidence interval crosses the null, consistent with the non-significant result (RR = 1.43, 95% CI 0.51–4.01; P = 0.50), and these results should therefore be interpreted with considerable caution.

#### 3.4.3 Disease-free survival

##### 3.4.3.1 Five-year DFS

Two studies (Wang et al., 2019; Chitapanarux et al., 2019) involving 2,450 patients were included in the analysis of 5-year disease-free survival. A total of 1,069 patients received conventional fractionation and 1,381 received hypofractionated radiotherapy. The number of events was 775 in the conventional fractionation group and 991 in the hypofractionated group.

Pooled analysis using the Mantel–Haenszel fixed-effect model demonstrated no statistically significant difference between treatment groups (*RR = 1.01, 95% CI 0.96–1.06; P = 0.83).* Statistical heterogeneity was low to moderate and not statistically significant (*χ² = 1.52, df = 1, P = 0.22; I² = 34%*).

**Figure 6:**
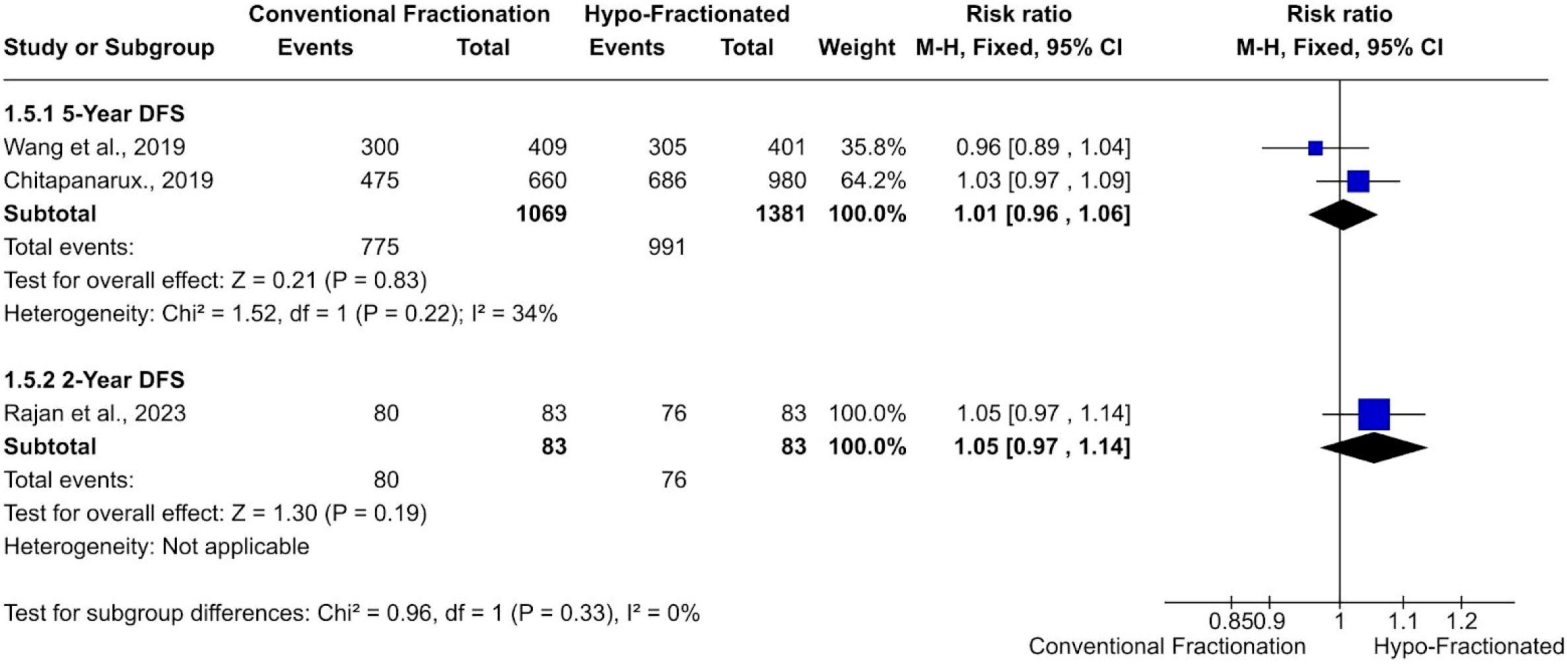
Forest plots illustrate the analysis of disease-free survival (DFS).

The forest plot (**Figure 6**) demonstrates closely aligned effect estimates between the two included studies, with narrow individual confidence intervals and a pooled diamond centered near the null, consistent with low-to-moderate heterogeneity (I² = 34%) and confirming the absence of any meaningful difference in 5-year disease-free survival between HFRT and CFRT.

## 4 Discussions

Because of better treatment and early identification, breast cancer is still one of the most common cancers in women, and its fatality rate is declining (1, 19). After breast-conserving surgery, radiation oncology is essential for locoregional management and increasing survival (3). This systematic review and meta-analysis compared HFRT and conventional CFRT in terms of DFS and early and late treatment-related side effects.

Our meta-analysis, which included 8 studies encompassing 5,495 patients, suggested a lower risk of acute radiation dermatitis in the hypofractionated group; however, this difference was not statistically significant under the random-effects model. For other outcomes, including lymphedema, and disease-free survival, no statistically significant differences were observed between the two treatment groups. These findings suggest that HFRT is a viable alternative to CFRT, offering comparable oncological outcomes while potentially minimizing treatment-related toxicity, particularly in terms of acute skin reactions.

A key observation from our meta-analysis was the significantly higher risk of acute radiation dermatitis in patients receiving conventional RT. This finding aligns with previous studies suggesting that prolonged treatment duration and cumulative radiation dose distribution contribute to increased skin toxicity (20, 21). For example, Braund et al. reported that conventional fractionation regimens were associated with a higher incidence of acute skin reactions compared to hypofractionated protocols, highlighting the benefits of shorter treatment schedules in reducing radiation-induced skin damage (22).

Beyond the statistical findings, it is crucial to consider the practical implications of these results for clinical practice. Our meta-analysis suggests that hypofractionated radiotherapy (HFRT) may be particularly beneficial for patients with early-stage, node-negative breast cancer, as supported by the DBCG HYPO trial (15). The comparable oncological outcomes and reduced risk of acute radiation dermatitis make HFRT an attractive option for this population. Additionally, the shorter treatment duration of HFRT can alleviate the burden on patients who may struggle with prolonged treatment schedules, such as those with limited access to healthcare facilities or significant comorbidities. Therefore, surgeons and oncologists should consider HFRT as a standard treatment option in appropriate cases, considering individual patient factors and preferences.

The heterogeneity observed in outcomes across studies may be attributed to variations in patient characteristics, radiation techniques, and follow-up durations. Differences in fractionation schedules, and treatment planning systems may have contributed to variability in reported toxicity and oncologic outcomes across studies. Furthermore, inconsistencies in outcome definitions and toxicity assessment methods across studies may have contributed to the variability observed in the results, underscoring the importance of standardized reporting in future studies. The substantial heterogeneity observed in some outcomes, particularly acute radiation dermatitis, further limits the certainty of pooled estimates and highlights the need for cautious interpretation.

Our analysis has several limitations. First, the lack of individual patient-level data limits the precision and granularity of our findings, as we relied on aggregate-level data from published studies. Second, variations in study design, including differences in radiation techniques, fractionation schedules, and toxicity assessment methodologies, introduce potential bias when comparing outcomes. Additionally, follow-up durations varied across studies, potentially affecting the assessment of long-term oncological outcomes. Lastly, the potential for publication bias, given the inclusion of only published studies, may lead to an overestimation of HFRT’s benefits. Future meta-analyses should incorporate unpublished data or trial registries to mitigate this risk.

Future research should focus on long-term outcomes, particularly chronic toxicities and patient-reported quality-of-life measures, to better understand the comparative benefits of HFRT and CFRT. Standardization of radiation protocols, toxicity grading criteria, and outcome definitions will enhance comparability and allow for more robust conclusions in future meta-analyses. Additionally, further investigations into patient-specific factors, such as age, tumor characteristics, and comorbidities, could help identify subgroups that would benefit most from personalized treatment approaches. Tailoring radiation regimens based on individual risk profiles could optimize clinical outcomes, improve patient tolerance, and enhance quality of life in breast cancer patients.

## 5 Conclusion

HFRT demonstrated comparable oncological outcomes to CFRT, with no clear increase in treatment-related toxicity across the analyzed outcomes. These findings support the use of HFRT as an effective and clinically practical alternative to conventional fractionation in patients with breast cancer. In particular, the comparable disease-free survival observed in this analysis reinforces the oncologic acceptability of shorter treatment schedules. Although toxicity findings were generally favorable, the heterogeneity among the included studies warrants cautious interpretation, particularly for outcomes with variable reporting across trials. Nevertheless, the overall evidence included in this review supports the continued integration of HFRT into contemporary breast cancer management. Future research should focus on standardized treatment protocols, consistent outcome reporting, long-term toxicity assessment, and patient-reported quality-of-life outcomes to better define the role of HFRT in clinical practice.

## Data Availability

The original contributions presented in the study are included in the article/supplementary material. Further inquiries can be directed to the corresponding author.
